# Retraction: Universal primers for HBV genome DNA amplification across subtypes: a case study for designing more effective viral primers

**DOI:** 10.1186/1743-422X-4-119

**Published:** 2007-10-31

**Authors:** Qingrun Zhang, Guanghua Wu, Elliott Richards, Shan'gang Jia, Changqing Zeng

**Affiliations:** 1 Beijing Institute of Genomics, Chinese Academy of Sciences, Beijing 101300, China; 2 Department of Biology, College of Biology and Agriculture, Brigham Young University, Provo UT 84602, USA

## Abstract

This is a retraction of the article submitted by Zhang et al. *Virology J *2007, **4**:92

## Retraction

Mr. Wu submitted this article [1] to *Virology Journal *on the assumption that the co-authors had agreed to the submission. Since this is not the case, the authors are retracting the article. Mr. Wu is deeply sorry for any inconvenience this may have caused to the editorial and publishing staff. An apology is also extended to the readers.
